# Sialylation: A Cloak for Tumors to Trick the Immune System in the Microenvironment

**DOI:** 10.3390/biology12060832

**Published:** 2023-06-08

**Authors:** Xiaoman Zhou, Kaijun Chi, Chairui Zhang, Quan Liu, Ganglong Yang

**Affiliations:** 1 The Key Laboratory of Carbohydrate Chemistry and Biotechnology, Ministry of Education, School of Biotechnology, Jiangnan University, Wuxi 214122, China; 2 Department of Medical Oncology, Affiliated Hospital of Jiangnan University, Wuxi 214122, China; 3 State Key Laboratory of Biochemical Engineering, Institute of Process Engineering, Chinese Academy of Sciences, Beijing 100190, China

**Keywords:** sialylation, Siglecs, immune checkpoint, tumors, tumor microenvironment

## Abstract

**Simple Summary:**

Tumor cells accumulate sialylation in tissues via the coordination of sialyltransferases and sialidases. Tumor sialylation can block receptor and ligand binding at a physical level and actively inhibit immune activation by binding to the Siglec receptor of immune cells, creating an immunosuppressive microenvironment. Blocking the sialylation–Siglec axis in tumor tissues could alleviate the suppression of the immune microenvironment.

**Abstract:**

The tumor microenvironment (TME), where the tumor cells incite the surrounding normal cells to create an immune suppressive environment, reduces the effectiveness of immune responses during cancer development. Sialylation, a type of glycosylation that occurs on cell surface proteins, lipids, and glycoRNAs, is known to accumulate in tumors and acts as a “cloak” to help tumor cells evade immunological surveillance. In the last few years, the role of sialylation in tumor proliferation and metastasis has become increasingly evident. With the advent of single-cell and spatial sequencing technologies, more research is being conducted to understand the effects of sialylation on immunity regulation. This review provides updated insights into recent research on the function of sialylation in tumor biology and summarizes the latest developments in sialylation-targeted tumor therapeutics, including antibody-mediated and metabolic-based sialylation inhibition, as well as interference with sialic acid–Siglec interaction.

## 1. Introduction

Sialic acid is a group of negatively charged nine-carbon monosaccharides, which are extensively distributed throughout the cell membranes of most vertebrate and higher invertebrate organisms [1]. Sialylation, a form of glycosylation featuring sialic acid at the terminal, is crucial for a range of physiological processes, such as cell signaling, cell adhesion, and immunomodulation, as it connects cells with extracellular environments [2,3,4].

As described in our previous paper [3], the metabolic pathway of sialic acid is facilitated by a series of enzymatic reactions that catalyze the synthesis, activation, and transfer of sialic acids to glycoconjugates [5,6]. Notably, the cytidine monophosphate N-acetylneuraminic acid (CMP-Neu5Ac), the product of sialic acid biosynthesis, is the only activated donor for sialylation in constructing cell surface molecules, such as glycoproteins, glycolipids, gangliosides, and even glycoRNAs. Sialyltransferases, a group of catalytic enzymes responsible for the addition of sialic acid from CMP-Neu5Ac to glycans in the Golgi apparatus, play a central role in regulating cellular sialylation [7]. Sialidases, on the other hand, are primarily responsible for removing sialic acid from glycans. Mammalian sialidases only have four types, NEU1-4, which are found in different intracellular compartments and negatively affect the level of cell surface sialylation, thereby preserving sialylation homeostasis [8,9,10,11]. Normal cells can precisely regulate sialic acid synthesis and degradation processes. However, in tumors, there is an accumulation of sialylation due to the high expression of sialyltransferases and the altered expression of sialidases [12].

The tumor microenvironment (TME) comprises various cellular and acellular components that collectively promote tumor proliferation, invasion, metastasis, and response to therapies [13]. In this ecosystem, tumor cells evade the surveillance of the immune system by counteracting the surrounding macrophages, fibroblasts, and T regulatory cells, as well as by suppressing the activity of immune cells via specific critical immunoregulatory pathways, such as immune checkpoints. Over the past decade, cancer immunotherapy, particularly the application of immune checkpoint inhibitors, has significantly advanced cancer treatment. The use of inhibitors targeting immune checkpoint receptors, such as PD-1 and CTLA-4, has notably enhanced patient outcomes, enabling long-term tumor stabilization and even remission. However, for some tumors, such as melanoma, the treatment response rate was less than half, suggesting that the immune escape of tumor cells depends not only on immune checkpoints, such as CTLA-4 and PD-1/PD-L1 but also other immunosuppressive modalities [14,15].

Recent studies have revealed that sialic acid is regarded as a ligand for Siglecs, another group of immune checkpoint proteins. Sialylation–Siglecs interactions enable high tumor sialylation to inhibit the functions of some immune cells, causing immune escape. Therefore, there has been a growing interest in targeting sialic acid for tumor therapy. Sialic-acid-targeting therapies are designed to exploit the high expression of sialic acid on the surface of tumor cells, which can lead to the selective killing of these cells. Several strategies are being explored to target sialic acid, including antibodies, small molecules, and enzyme-mediated approaches [16]. These therapies have the potential to target tumor cells while leaving low-sialylated cells untouched, improving the efficacy of cancer treatments and reducing unwanted side effects.

This review is a follow-up to our previous review of sialylation function in tumors [3], updated with the latest research on sialylation and its regulation of the immune system. We summarized the recent findings on sialylation’s precise regulation via sialyltransferases and sialidases and discussed how sialylation influences immune cell behavior towards tumors. Additionally, we also highlighted novel approaches to block the sialylation–Siglecs axis as a potential immunotherapeutic to boost anti-tumor immunity and potentially curtail cancer progression. We hope that this review will provide a brief overview for researchers wishing to study the role of sialic acid in tumor microenvironments.

## 2. Sialylation Accumulates in Cancer Tissues and Promotes Tumor Development

The biosynthesis of glycan is an orderly, stepwise addition reaction and is strictly regulated in the cell. Changes in the physiology or environment of cells or tissues can cause alterations in the glycan modification process, providing a basis for investigating mechanisms and diagnosing diseases [17,18]. As sialylation takes place in a later stage of glycan processing in Golgi, the synthesis of sialic-acid-modified glycoconjugates is not only affected by the preparation stage of the glycan backbone but is also governed by its own specific regulatory network. Next, we discuss the characteristic highly expressed sialylated glycan structures present in the tumor microenvironment, as well as the enzyme families that participate in sialylation.

### 2.1. Typical Sialylated Glycans in Tumors

**Figure 1:**
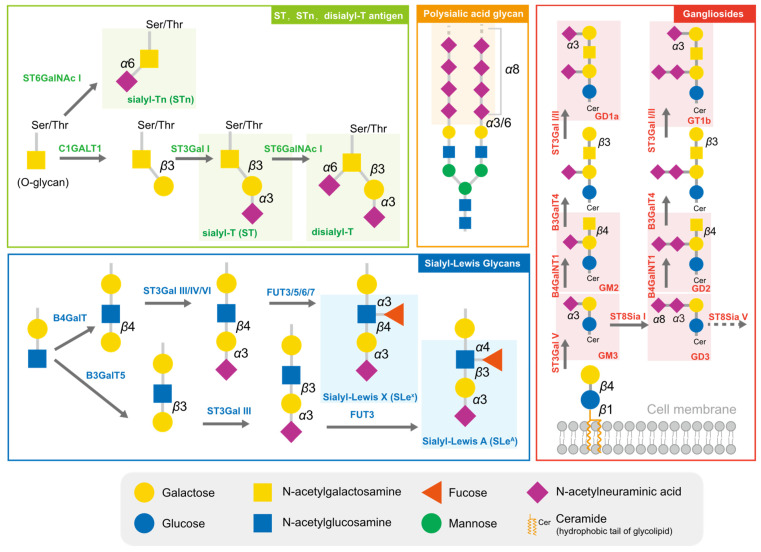
The typical sialylated glycans in tumors. The sialylated glycan structures typically highly expressed in tumors are marked with the translucent rectangular box.

Cancer cells are cloaked by a plethora of glycosylation, with many of them exhibiting a high level of sialylation. Glycosylation has the potential to mask essential antigenic and receptor-binding sites, as well as interact with certain checkpoints, enabling cells to avoid being recognized by enemy-identifying signals [19,20]. In addition to physical shielding, certain glycan structures possess specific physiological functions; they can act as ligands to mediate signal recognition and immune suppression [21]. These glycans comprise sialyl-Tn (STn), sialyl-T (ST), disialyl-T, sialyl-Lewis antigens, polysialic acid, and gangliosides (Figure 1). The broad distribution and advantageous effects on cancer cells have rendered sialylated glycans a hallmark of cancer [22].

The short sialylated O-glycan STn and ST antigens are aberrantly expressed in several cancers, with high levels being mainly observed in carcinomas and associated with aggressive tumors, such as those with chemotherapy resistance and poor prognosis [23,24]. The increased level of STn in tumor cells is primarily attributed to the upregulation of ppGalNAcT and ST6GalNAc I, combined with the low efficiency of COSMC, which assists C1GALT1 in extending glycans [24]. High serum concentrations of STn have been observed in patients with breast cancer [23], prostate cancer [24], bladder cancer [25], cervical cancer [26], and ovarian cancers and correlated to tumor grade and metastasis [27]. Due to the high prevalence of STn antigen expression in tumors, serum STn levels can be clinically utilized to assist with tumor diagnosis. ST antigens were also elevated in cancer, and the silencing of *ST3GAL1* significantly reduced the level of these antigens, further reducing the tumor size in the prostate cancer xenograft mouse model [28]. 

Sialyl-Lewis (SLe) antigens, SLe^A^ and SLe^X^, are other typical structures of sialyl glycans and act as ligands for selectins, a family of lectins involved in lymphocyte trafficking. Cancer cells that are disguised by SLe^A^ and SLe^X^ are mistaken for leucocytes during their epithelial–mesenchymal transition and travel throughout the circulation system [29]. E-selectins, the primary receptors of SLe^A^ and SLe^X^, are adhesion molecules required for leukocyte recruitment during the early stages of inflammation. The accumulation of SLe^A^ and SLe^X^ leads to cell adhesion and subsequent trans endothelial migration of traveling tumor cells. In ovarian tumors, mucins MUC16 and MUC1 are major carriers of SLe^A^ and SLe^x^ and are used as biomarkers [30].

Polysialic acid is a polymer of sialic acid, with α2,8 and α2,9 linkages and a length ranging from 8 to 400 units. It is a crucial glycosylation type for several essential proteins, providing them with a negative charge. In mammalian cells, three sialyltransferases (ST8Sia II, ST8Sia III, and ST8Sia IV) are responsible for the extension of polysialic acid glycans [31]. Polysialic acid is often detected in brain tissues and is also found in immune cells. Several molecules are found to undergo polysialylation, such as neural cell adhesion molecules (NCAMs) [32,33], chemokine receptor 7 (CCR7) [34,35], CD36 [36], and E-selectin ligand 1 (ESL-1) [37]. Interestingly, the sialyltransferase ST8Sia II and ST8Sia III can polysialylate independently. These polysialylated glycoproteins participate in physiological processes, including cell adhesion between cells and cells with a matrix, cell migration, synapse formation, and the functional plasticity of the developing nervous system. In tumor cells, the level of polysialic acid chains correlates with an aggressive phenotype and the resistance of cancer treatment [38]. 

Glycolipids, molecules composed of one or more carbohydrate residues linked to a hydrophobic lipid moiety via a β-glycosidic linkage, are mainly found in lipid rafts on the outer leaflet of the plasma membrane bilayer. Some gangliosides (GD2, GD3, GM2, GM3, fucosyl-GM1) are distinguishable from other gangliosides via their significantly lower or absent expression in normal cells, yet they are highly expressed in tumor cells [39]. GD3 and GD2 are highly expressed in certain tumors and can be utilized as targets for immunotherapy. However, they can also inhibit the function of immune cells, such as macrophages, via binding to Siglec-7 [40]. 

### 2.2. Sialyltransferases Are Critical Enzymes for Hypersialylation

Approximately twenty sialyltransferases are involved in the positive regulation of human cell sialylation, and these sialyltransferases are classified into four types based on the differences in their substrate structure and the linkage of the formed sialylation: ST3Gal I-VI (add Neu5Ac to galactose in an α2,3 linkage); ST6Gal I-II (add Neu5Ac to galactose in an α2,6 linkage); ST6GalNAc I-VI (add Neu5Ac to GalNAc in an α2,6 linkage); and ST8Sia I-VI (add Neu5Ac to Neu5Ac in an α2,8 or α2,9 linkage) [7]. The increased modification of sialic acid in various malignant tumors is caused by the high expression of sialyltransferases. Hypersialylation in the tumor microenvironment alters its physiological characteristics, blocking some immunological recognition and communication [41]. More importantly, sialylation also promotes tumor cell survival and drug resistance, as well as suppressing surrounding immune cells, which helps tumor cells to survive [42]. Next, we briefly summarized recent research on sialyltransferases in tumors.

The sialyltransferase ST6Gal I is a well-studied enzyme, catalyzing the addition of Neu5Ac to galactose residues of Gal β1-4 GlcNAc in an α2,6 bond mainly on N-glycans. Elevated levels of ST6Gal I have been linked to a number of different cancer types and can be a driver of malignant progression, as well as resistance to therapy [43,44,45,46,47,48,49]. This is further supported by the pancreas-specific genetic deletion of *ST6GAL1* in a mouse model, which delays cancer formation [50]. Additionally, ST6Gal I has been observed to add sialylation in cell surface receptors, such as PDGFRB [44], EGFR [51,52], and PECAM [53], which increases protein levels and phosphorylation to stimulate pathways such as PI3K/AKT and RAS [43], thus contributing to tumor growth. ST6Gal I has also been implicated in the immune evasion in hepatocarcinoma cells, where it increases levels of MMP9 and suppresses T-cell proliferation [54]. Moreover, ST6Gal I is released into the extracellular milieu in either exosome or free forms, thereby remodeling cell surface and secreted glycans, which has been linked to aggressive tumor cell proliferation in breast cancer [55]. ST6Gal II is another enzyme that can add α2,6-linked sialic acid to N-glycans. Nonetheless, this enzyme is predominately expressed in the embryonic and perinatal stages of brain tissue [56]. In a recent study of breast cancer, ST6Gal II accumulated in tumor tissue and was associated with tumor malignancy. The inhibition of ST6Gal II caused the downregulation of cell adhesion and invasion-associated proteins, resulting in reduced tumor migration [57]. Similarly, silencing *ST6GAL2* in a follicular thyroid carcinoma reduced tumor growth by inactivating the Hippo pathway in an in vivo model [58].

The ST3Gal family, consisting of six members (ST3Gal I-VI), facilitates the transfer of sialic acid to the terminal galactose residues of glycochains via an α2,3-linkage in both glycoproteins and glycolipids. ST3Gal I, which predominantly functions in core-1 O-glycans, catalyzes the transfer of Neu5Ac to a galactose residue in an α2,3 bond to produce sialyl-T antigen. The upregulation of ST3Gal I has been reported in many malignant tissues, such as ovarian cancer [59], glioblastoma tumors [60], and melanomas [61], and it has been associated with tumorigenesis, poor clinical outcomes and an inflammatory phenotype. Additionally, CD55, an essential immune checkpoint molecule, has been reported to be O-glycosylated by ST3Gal I to help cancer cells escape immune attack [62]. 

ST3Gal II, in contrast, prefers gangliosides as its substrate to form GD1a and GT1b [63,64]. The elevated expression of ST3Gal II has been associated with advanced stages of cancer and poor clinical outcomes. In addition, ST3Gal II is the only enzyme responsible for synthesizing the glycosphingolipid SSEA4, a well-known biomarker of several cancers [65,66]. Furthermore, *ST3GAL2* knockdown led to a dramatic growth reduction in colorectal cancer in xenografted mice models [67].

ST3Gal III, ST3Gal IV, and ST3Gal VI are implicated in the formation of SLe^A^ and SLe^X^ glycans on the cell surface, which act as binding ligands for selectins and are essential for metastasis [68,69]. The high expression of ST3Gal III has a strong positive correlation with poor prognosis in gastric cancer [70], and ST3Gal IV is the main enzyme for generating ligands of Siglec-7 and -9 [71,72]. ST3Gal VI generates selectin ligands and accumulates in liver and urinary bladder cancers [73,74]. Moreover, *ST3GAL5* encodes GM3 synthase, the rate-limiting enzyme for the production of downstream gangliosides, and is, therefore, crucial to gangliosides synthesis [63]. In renal cell carcinoma research, *ST3GAL5* was consistently overexpressed in tumor tissue and correlated with the infiltration of exhausted CD8^+^ T cells, indicating that ST3Gal V contributes to immune suppression [75]. 

The ST6GalNAc family, consisting of six members, ST6GalNAc I-VI, catalyzes the α2,6 glycosidic linkage of Neu5Ac to the GalNAc residues on O-glycans or glycolipids. ST6GalNAc I, which adds Neu5Ac to O-linked GalNAc residues to form sialyl-Tn (STn), is particularly significant [76]. Evidence suggests that the overexpression of STn is associated with poor clinical prognosis in a wide range of cancer types [27,77], making it a well-known tumor-associated carbohydrate antigen. One functional study found that ST6GalNAc I can promote tumor growth and metastasis and is related to cancer cell stemness [78]. Furthermore, cytokines such as IL-13 promote the phosphorylation of STAT6, which, in turn, activates the transcription of *ST6GALNAC1*, thereby facilitating the formation of STn [79]. The STn inhibits T-cell responses by binding to Siglec-15, leading to immune evasion in the tumor microenvironment [80,81].

ST6GalNAc II is an enzyme responsible for synthesizing ST and STn antigens. The role of ST6GalNAc II in tumors varies with the stage and status of the tumor. In breast cancer metastasis, ST6GalNAc II catalyzes the formation of ST and STn that blocks tumor binding to galectin, negatively affecting tumor metastasis [82,83]. However, in the tumor microenvironment, ST6GalNAc II is positively correlated with higher tumor stage and worse prognosis [84]. 

Another ST6GalNAc family member for O-glycan is ST6GalNAc IV, a key ST6GalNAc enzyme that is involved in the formation of disialyl-T antigen and GD1α from sialyl-lactotetraosyl-ceramide GM1b (gangliosides). In a primary lung cancer model, the upregulation of *ST6GALNAC4* was demonstrated to confer glycosylation changes in tumor cells, contributing to their metastatic activity. This is likely due to the preservation of the T-antigen presentation and adherence to galectin 3 [85]. The catalytic product of ST6GalNAc IV, the disialyl-T antigen, was shown to be a ligand for Siglec-7. The high expression of ST6GalNAc IV increased disialyl-T antigens in CD162 and CD45 and inhibited NK cell activity via the binding of Siglec-7 in chronic lymphocytic leukemia B cells [86]. Moreover, in liver cancer, the elevated *ST6GALNAC4* promoted tumor proliferation, migration and invasion ability, and affected the expression of immune checkpoints on tumor cells [87].

The sialyltransferases ST6GalNAc III, ST6GalNAc V, and ST6GalNAc VI are mainly involved in glycolipid synthesis. ST6GalNAc III and ST6GalNAc V use GM1b as a substrate to synthesize GD1α [88,89], whereas ST6GalNAc VI catalyzes the synthesis of α-series gangliosides, including GD1α, GT1aα, and GQ1bα; globo-series glycosphingolipids (GSL); and disialyl Le^A^. It has been reported that ST6GalNAc III increased M2 macrophages via the accumulation of prostaglandin and arachidonic acid in gastric cancer [90]. *ST6GALNAC5* was expressed at low levels in tumors, and its overexpression significantly inhibited tumor growth and invasiveness [91]. On the other hand, the downregulation of *ST6GALNAC6* resulted in a change from disialyl Le^A^ to sialyl Le^A^ and an elevation in E-selectin binding activity during metastasis, which supports inflammation-driven carcinogenesis by reducing its binding to the immunoregulatory Siglec-7 [92].

Members of the ST8Sia family catalyze the transfer of sialic acid to another sialic acid, forming α2,8 linkages. Notably, the 2,8-disialic glycan structure, ligands for Siglec-7 and Siglec-9, can potentially regulate immune responses. Notably, ST8Sia I, also known as GD3 synthase, is positively correlated with the astrocytoma grade and accumulates in glioblastomas [93]. Similarly, ST8Sia II and ST8Sia IV are polysialyltransferases that produce polysialylated cell adhesion molecules, which are highly expressed during cancer development. In tumors, the expression of *ST8SIA2* has been shown to correlate with the tumor stage [94]. Moreover, the overexpression of *ST8SIA2* increased the invasiveness and metastatic abilities of small-cell lung cancer cells in vitro [94,95]. Additionally, *ST8SIA4* is overexpressed in breast cancer tissues and contributes to chemoresistance in acute myeloid leukemia [96,97]. Furthermore, ST8Sia III, which causes the sialylation of a variety of glycolipids (GM3, GD3, and α2,3-sialylparagloboside), was identified as a therapeutic target for glioblastomas [98]. Other ST8Sia family members, such as ST8Sia V and ST8Sia VI, are also related to malignant potential. ST8Sia V, the enzyme adding Neu5Ac to gangliosides, was expressed at a low level and negatively correlated with patient survival in bladder cancer and colon cancer [99,100]. ST8Sia VI generates disialic acid structures preferentially on O-linked glycoproteins, and these products are proven to bind with Siglec-7 and Siglec-9. Studies have shown that ST8Sia VI contributes to tumor growth in a mouse model by inhibiting immune responses via the alteration of the macrophage polarization towards M2 and increasing the immune modulator arginase in the tumor microenvironment [101].

### 2.3. The Function of Sialidases in Tumor Sialylation Regulation

The sialidases and sialyltransferases in cells collectively act to maintain sialylation homeostasis. In tumor cells, an abnormally increased level of sialylation is generally attributed to elevated sialyltransferase activities; however, the role of sialidases in regulating the sialylation levels remains to be addressed.

Mammalian sialidases, NEU1-4, are enzymes with distinct cellular localizations. NEU1, mainly present in lysosomes, is associated with the degradation of sialylated glycans and the recycling of sialic acid. Evidence suggests that the upregulation of NEU1 in cancers may increase the utilization of sialic acid, thus contributing to the maintenance of cell sialylation. NEU1 is highly expressed in various cancers, such as liver cancer [102], pancreatic cancer [103], ovarian cancer [104], and melanoma [105]. However, NEU1 is reported to be expressed at low levels in certain stages of tumors and has been found to remove cytosolic sialic acid modifications and inhibit tumor progression [10,106]. Therefore, the effect of NEU1 on tumors must be comprehensively and dialectically analyzed. NEU2 is located in the cytosol and predominantly inhibits tumor growth. The decrease in NEU2 leads to increased sialylation levels and reduces the stemness-like properties of cancer stem cells [107]. Additionally, NEU2 causes a reduction in α2,6-linked sialylation on the Fas protein, leading to apoptosis in pancreatic cancer [108]. In ovarian cancer cells, the overexpression of NEU2 leads to a significant reduction in α2,3- and α2,6-linked sialylation and induces cellular autophagy by upregulating the expression of ATG5, an essential protein involved in autophagosome formation [109]. NEU3, a membrane sialidase, is essential for the hydrolysis of sialic acid in ganglioside. In colon cancer, the upregulation of NEU3 accumulates lactosylceramide and leads to protection against programmed cell death [110]. NEU4 is located in the ER membrane, mitochondria and lysosomes, and is downregulated in many tumors. NEU4 has been reported to negatively regulate the motility of tumors via the desialylation of CD44 in hepatocellular carcinoma [111], as well as reduce sialyl Lewis antigens to prevent cell adhesion to E-selectin in colon cancer [112]. While the role of sialidases in tumors may vary based on tumor type and status, tumor cells consistently regulate sialidase expression and control sialylation in a way that promotes tumor progression.

Sialyltransferases and sialidases are strictly and dynamically regulated to increase and maintain high sialylation levels, which helps to induce the immunosuppressive status of the tumor microenvironment via interactions with immune cells, thus facilitating tumor survival and growth. In the following sections, we summarize the latest research progress regarding the mutual interactions between sialylation and immunity in recent years.

## 3. Sialylation Reshapes the Tumor Microenvironment

### 3.1. Sialylation Serves as a Camouflage for the Passive Protection of Tumor Cells

In recent years, knowledge on the distribution and visualization of sialylation in tumor microenvironments has increased due to the development of multiple sialic acid imaging technologies. A study on ovarian cancer utilized a mass spectrometry imaging technique with sialic acid derivation and revealed a significant increase in α2,3- and α2,6-linked sialylation in both cancer tissues and adjacent stromal tissues [113]. In pancreatic cancer research, mass spectrometry imaging was used to detect an increase in sialylated N-glycans in all cancer tissues, and the characteristic tetrasaccharide structure of CA19-9 was also enriched in these tissues [114]. In addition to the mass spectrometry imaging technique, the sialic-acid-specific labeling technique has been widely used to visualize sialylation in tumor microenvironments. A sialic-acid-specific quantum dot was utilized for the efficient and selective labeling of sialic acid, and the staining results of cancerous tissue slices demonstrated strong fluorescence signals in cancerous tissues, with a clear boundary to distinguish them from normal tissues [115]. These findings supported the notion of a substantial accumulation of sialylation in the tumor microenvironment. With newer technologies applied to the visualization of sialylation, the distribution of sialic acid in tumor microenvironments can be elucidated. The glycans on the cell surface, especially sialylated glycans, have a significant protective effect and are considered as cloaks of the cell. Reducing glycan synthesis using chemicals such as a glucose analog (2-deoxy-d-glucose) could efficiently rescue the activity of CAR-T cells in xenograft mouse models of pancreatic adenocarcinoma [116]. Due to the unique physicochemical properties and the terminal location on glycans, sialic acid is particularly suitable for covering key recognition sites to block the recognition of related receptors and specific antibodies [117]. In this sense, cancer cells evade some signal regulation and antibody-dependent immune attacks.

The presence of sialylation on the surface of tumor cells enhances their ability to evade apoptosis upon encountering immune cells that release apoptotic signals. The hypersialylation of the Fas receptor (FasR) and tumor necrosis factor receptor (TNFR1) has been observed to hinder the internalization of these receptors and decrease downstream cell death signaling in tumor cells [118]. Additionally, galectins, a family of lectins that recognize β-galactosides via their conserved carbohydrate recognition domains, have been found to induce cell apoptosis [119]. However, the sialylation of extracellular galectin-binding partners, such as integrins, can disrupt galactose–galectin interactions and mitigate galectin-induced apoptotic signaling [42,120,121,122,123]. In this sense, tumor cells survive under immune attack. 

Aside from passive defense, sialylation also plays a vital role in actively modulating the activity of surrounding immune cells, a field that has been widely studied in recent years [124]. In particular, the sialylated glycan has specific receptors in mammals, and, in the following section, we discuss its interactions with ligands in the tumor microenvironment.

### 3.2. Tumor Cells Deceive Immune Cells through the Binding of Sialylated Ligands to Siglecs

The environment in which a tumor grows is a complex social milieu, where tumor cells must not only exchange material and energy with the external environment but also integrate into the “social networks” of surrounding cells, engaging in mutually beneficial relationships. Based on the infiltration of immune cells in tumor tissues, the tumor microenvironment can be simply categorized into two distinct types: inflamed and non-inflamed. These types are commonly referred to as “hot” and “cold” tumors, respectively [125]. In hot tumors, tumor cells establish contact with immune cells. However, they hinder the functioning of immune cells by employing various pathways, such as immune checkpoints [126]. Remarkably, sialylated glycans are believed to possess immunosuppressive properties. The interaction between sialylation on tumor cells and the sialic acid receptors (Siglecs) on immune cells conveys immunosuppressive signals, thus avoiding eliciting an inflammatory response from the immune cells [127,128].

**Figure 2:**
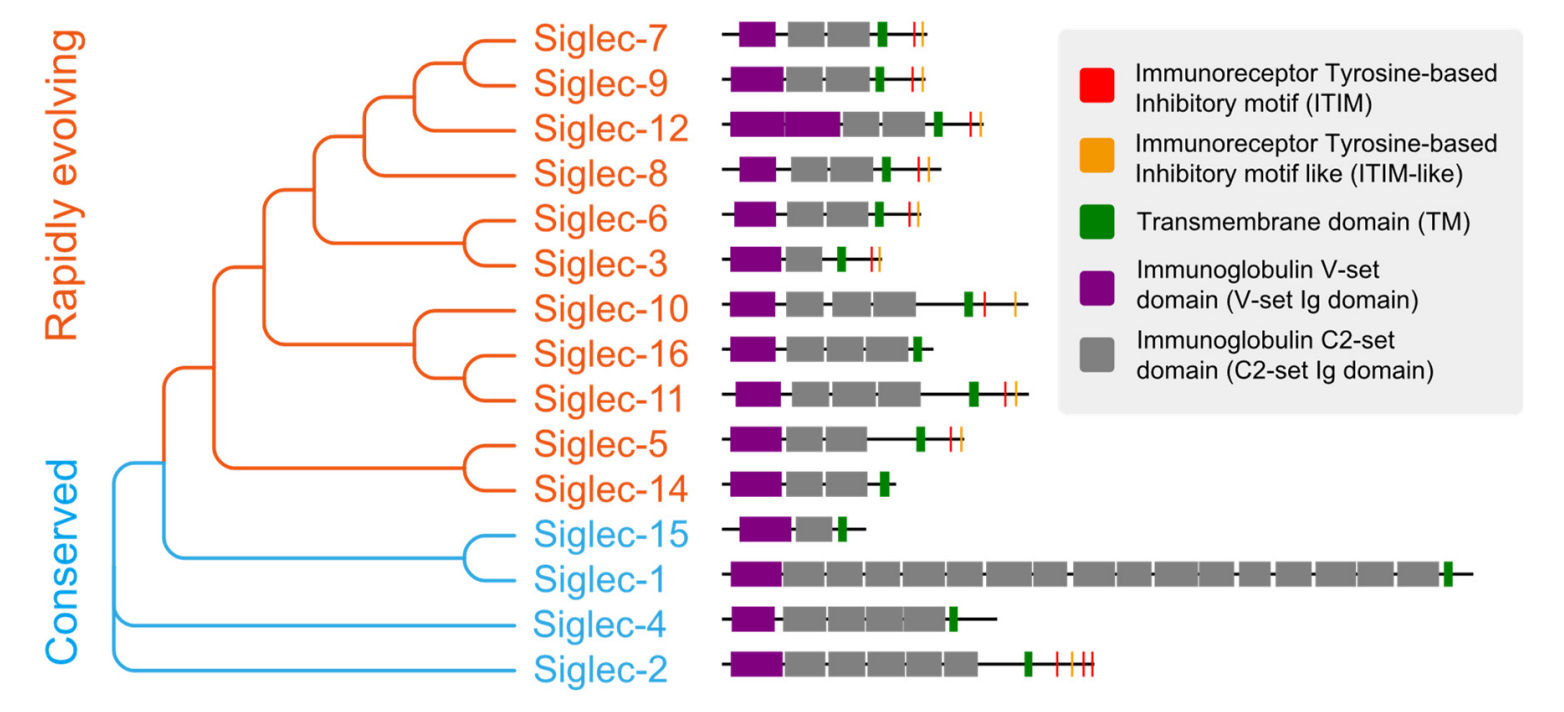
The evolutionary tree and protein structure of human Siglecs. The order of Siglecs is based on sequence similarity and the sequences are displayed according to annotation.

**Figure 3:**
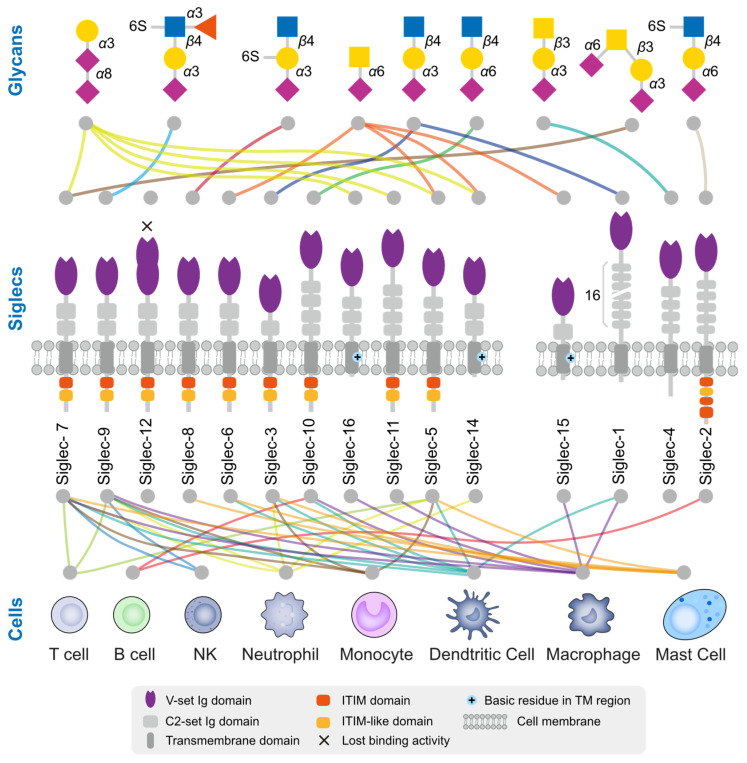
Ligand glycan structure and expression cells of human Siglecs. There exists a total of 15 Siglecs within the human body, among which Siglec-14 has incurred a mutation resulting in the loss of its sialic acid binding functionality. The Siglecs are arranged based on their sequence similarity. The upper section provides insight into the preference of Siglec for glycan structures, while the lower section indicates the immune cells that have been reported to predominantly express these Siglecs. The colors of the lines are distinguished based on the glycan structure or cell type.

Siglecs are a family of receptors that bind to sialic-acid-containing glycans (sialylated glycans) [129]. Thus far, fifteen Siglecs have been identified in human cells. Interestingly, Siglec-12 (also named Siglec-XII) lost the ability to bind sialic-acid-containing glycans due to a homozygous missense mutation that was found only in humans [130]. We arranged and grouped the Siglecs based on the similarity of their protein sequences in human cells (Figure 2), and the results clearly demonstrated that all of these Siglecs had a conserved transmembrane domain (TM) and N-terminal immunoglobulin V-set domain (V-set Ig domain). The V-set Ig domain contained the arginine active site with sialic-acid-binding activity. Siglecs also had different numbers of immunoglobulin C2-set domains (C2-set Ig domain) that support the binding domain and are all characterized by a single transmembrane structure, indicating their location to be primarily on the cell membrane (Figure 3). For the intracellular part, nine (Siglec-2,3,5-11) Siglecs contained tyrosine-based inhibitory motifs (ITIM) that transmitted immunosuppressive signals via the engagement of SHP1 and SHP2 phosphatases upon ligand binding, three (Siglec-14,15,16) contained positive charges in the transmembrane region and can bind DAP12 to transmit immune activation signals, and the remaining two (Siglec-1,4) did not have signaling functions. Based on evolutionary similarity, Siglecs can be classified into conservative (Siglec-1, 2, 4, 15) and rapidly evolving types (Siglec-3, 5-12, 14, 16, also known as CD33-related types). Although the binding domain is similar, the ligand structures for those Siglecs differ (Figure 3) [21,131]. Next, we describe how tumors actively suppress the function of immune cells via Siglecs.

NK cells are crucial innate immune cells that are capable of killing tumor cells without prior antigen stimulation and are involved in critical functions such as cell lysis, secretion of chemokines, and cytokines to attract other immune cells. Tumor cells mitigate the threat of NK cells mainly via ligand binding to Siglec-7 and -9. Siglec-7/9 are primarily expressed in NK cells. Siglec-9 expression occurs during the early stages of NK cell differentiation [132], whereas Siglec-7 is predominantly expressed in highly cytotoxic mature NK cells [133]. Both Siglecs play a suppressive role in regulating immune homeostasis via the negative regulation of NK cell activation and reducing toxicity towards target cells. Siglec-7 and Siglec-9 are two of the most extensively studied members of the Siglec family. They share a high degree of similarity in their amino acid sequences, reaching up to 98%. However, they exhibit significant differences in their ligand preferences. Siglec-7 shows a higher affinity for α2,8-linked sialic acid and poor affinity for α2,3- and α2,6-linked sialic acids, primarily binding to O-glycans on mucin substrates such as CD43 [134,135,136]. Conversely, Siglec-9 exhibits a preference for α2,3- and α2,6-linked sialic acids in both N-glycans and O-glycans, mainly binding to sulfated SLe^x^ [137]. In tumor cells, the gangliosides DSGb5 and GD3 were reported to inhibit the cytotoxic activity of NK cells via binding to Siglec-7 on the surface of the NK cells [138,139]. Interaction experiments confirmed that PSGL-1 is a ligand for Siglec-7 on NK cells in multiple myeloma, and the treatment of myeloma cells with sialidase relieves the inhibition of NK cell activity and enhances the sensitivity of targeted drugs [140]. In another study on chronic lymphocytic leukemia, the disialyl-T antigen on CD162 and CD45 was proven to inhibit NK cells via binding to Siglec-7 [141]. Adenocarcinoma tissues exhibited high expression levels of MUC1 and MUC16, which contained substantial amounts of SLe^x^. The SLe^x^ shields tumors from immune attacks by binding to Siglec-9 on the surface of NK cells and conveying inhibitory signals [142].

The complex relationship between tumors and macrophages stems from the dual role of macrophages in tumor growth. As crucial components of the innate immune system, macrophages are responsible for the release of cytokines and the presentation of antigens. Macrophages can undergo different polarization states depending on environmental conditions and can be classified into two major groups: classically activated M1-type macrophages (pro-inflammatory, anti-tumor) and alternatively activated M2-type macrophages (anti-inflammatory, pro-tumor). In the interplay between tumor cells and macrophages, Siglec-7 and Siglec-9 remain the principal sialic acid receptors. Tumor cells upregulate the sialyltransferase ST3Gal IV, leading to an increase in ST antigen that inhibits macrophage activation and promotes macrophage differentiation in tumor-associated macrophages, in combination with Siglec-9, thereby inducing the formation of an immunosuppressive microenvironment [71]. Recent studies have also revealed that the high expression of *ST6GALNAC4*, which is driven by the oncogene *MYC*, promotes the synthesis of disialyl-T glycans in CD43. This process inhibits Siglec-7 and prevents macrophages from clearing tumors [143]. CD24 is also reported to inhibit macrophages vis its connection to Siglec-10, delivering the “do not eat me” signal. Blocking the binding between CD24 and Siglec-10 or reducing tumor CD24 expression through neutralizing antibodies can restore the ability of macrophages to inhibit tumor cells and improve animal survival [144].

Dendritic cells are the most potent and specialized antigen-presenting cells in the immune system. Recent studies have demonstrated increased expressions of Siglec-7, Siglec-9, and Siglec-10 in conventional DC cells derived from patient tumor samples, indicating their potential involvement in tumorigenesis. Additionally, transcriptomic and proteomic analyses revealed the impaired maturation of DC cells. The inhibition of these Siglecs in DC cells resulted in restored DC activity, leading to improved antigen uptake, processing, and T cell activation [145].

T cells are crucial players of adaptive immunity that mediate the killing of tumor cells and have become the focus of research on immune checkpoint and CAR-T therapy in recent years. While traditional immunotherapies are insufficient, the sialic acid–Siglec interactions are considered to provide a potential alternative immune checkpoint target. Generally, Siglec is not highly expressed in T cells, but Siglec-9 and Siglec-5 are abundantly expressed in tumor-infiltrating cytotoxic CD8^+^ T cells. Ligand binding to Siglec-9 strongly inhibits the TCR signaling pathway and essential effector functions of CD8^+^ T cells [146]. Similarly, Siglec-5 is highly expressed in a variety of T cells activated by antigens. Upon binding to ligands, highly expressed Siglec-5 inhibits the activation of the NFAT and AP-1 signaling pathways of T cell activation [147].

**Figure 4:**
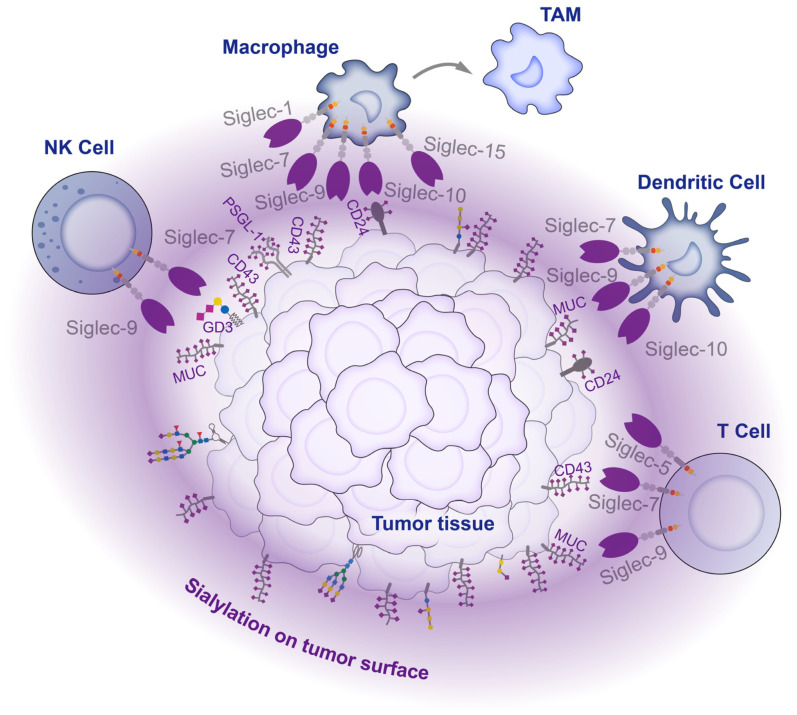
Interaction between tumor cells and immune cells in the tumor microenvironment. There are high levels of sialylation on the tumor cell surface. These sialylations act as ligands, engaging with Siglecs on infiltrating immune cells within the tumor tissue, inhibiting the activity of immune cells and affecting their differentiation, thereby mediating tumor immune escape.

Within the tumor microenvironment, tumors cells express glycosyltransferases that produce glycoproteins and glycolipids with typical glycan chains that bind to Siglecs, preventing immune cells from attacking the tumor. As a result, an immune-suppressive environment is created that supports tumor growth and invasion (Figure 4). However, despite the regulatory functions of most Siglecs in suppressing inflammation and modulating immune suppression, three specific Siglecs (Siglec-14, 15, and 16) have been identified to incite inflammation and stimulate immune activation [148,149,150]. The intricate mechanism underlying the interaction between sialylation and Siglecs in the tumor microenvironment necessitates further exploration.

## 4. Opportunities for Cancer Treatment via the Removal of Sialylation-Caused Immune Suppression

### 4.1. Metabolic Interference with Sialylation

The enhancement of sialic acid synthesis in the tumor microenvironment is a common phenomenon regarded as one of the hallmarks of cancer [22]. In previous studies, the metabolic network of sialic acid synthesis has been elucidated. In eukaryotic cells, sialic acid is synthesized in the cytoplasm and transported to the nucleus for cytidylate 5′-monophosphate (CMP)-Neu5Ac synthesis. Then, it is transported to the Golgi apparatus via the sialic acid transporter SLC35A1 and added to glycoconjugates via sialyltransferases before being secreted or delivered to the cell surface [3]. At this point, metabolic intervention to reduce tumor sialylation is considered to be a practical approach to block tumor sialylation.

**Figure 5:**
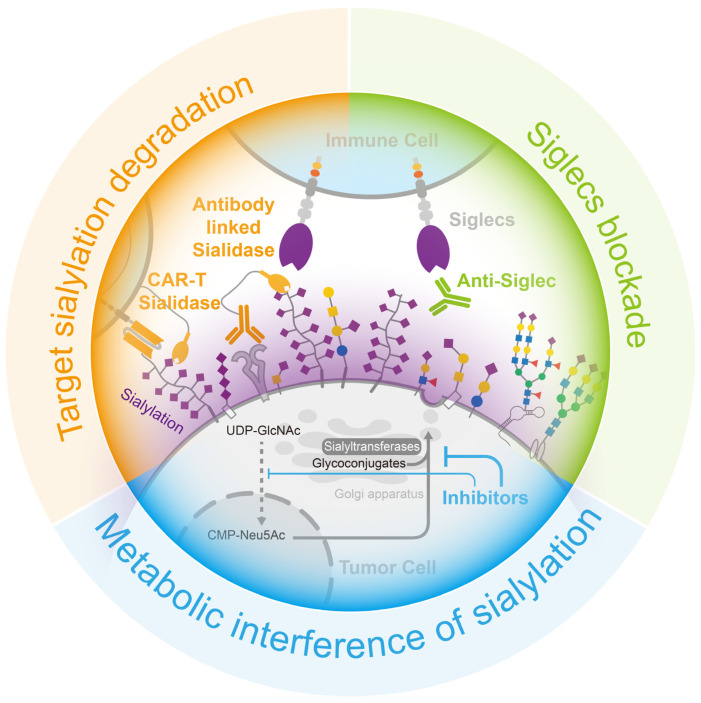
Opportunities for cancer treatment via the interruption of the sialylation–Siglecs axis. (Blue) the employment of inhibitors to interfere the formation of sialylation, (Green) the use of neutralizing antibodies to obstruct the binding of sialylation to Siglecs in immune cells, and (Orange) the targeted degradation of sialylation on the tumor cell surface through the use of sialidase-linked antibodies specifically directed to tumor cells.

Analogues of sialic acid can achieve a reduction in sialylation by sneaking into the sialic acid metabolic pathway and interfering with the function of sialyltransferase or sialidase, thereby constituting an efficient sialylation inhibitor of tumor cells (Figure 5). Among all the analogues, fluorinated sialic acid can provide long-term sialylation inhibition. Ac_5_3F_ax_-Neu5Ac acetylates, which enhances cellular uptake, and, when converted to CMP -3F_ax_-Neu5Ac, it also inhibits sialyltransferases, aided by an axial fluorine atom incorporated into the C-3 carbon [151]. Moreover, the accumulation of CMP-3F_ax_-Neu5Ac, an analogue of CMP-Neu5Ac, inhibits the activity of the GNE enzyme (the rate-limiting enzyme of sialic acid synthesis) via feedback regulation, thereby attenuating the cell’s own sialic acid synthesis and reducing sialylation [152]. In a study on pancreatic ductal adenocarcinoma, treatment with Ac_5_3FaxNeu5Ac resulted in the inhibition of the tumor cell production of α2,3-sialic acid and SLe^X^-modified glycans, leading to reduced adhesion of tumor cells to selectin. Subsequent animal experiments demonstrated that sialic acid inhibition significantly modified the composition of immune cells in the tumor microenvironment by increasing the proportion of CD8^+^ cells and NK cells, which facilitated the restoration of tumor immune surveillance [153]. However, the use of 3Fax-Neu5Ac as a global inhibitor of sialyltransferase may affect sialic acid metabolism in other normal organs and has been shown to be toxic to the liver and kidneys when used in large amounts in vivo [154]. This deficiency has been effectively addressed using nanoparticles coated with tumor-targeting antibodies [155] and self-assembled nanoparticles [156]. Innovation in drug delivery enables safer and more sustained in vivo inhibition of sialylation.

### 4.2. Target Sialylation Degradation

Sialidase is an enzyme that efficiently removes sialic acid from glycans. By coupling sialidase with tumor-targeting antibodies, the tumor-targeted removal of sialic acid can be achieved (Figure 5). HER2 is the marker protein of a breast cancer subtype, and several commercial antibody drugs are available for its treatment. 

A protein complex consisting of HER2 antibodies and sialidase from *Salmonella typhimurium* had been designed to precisely remove sialic acid from the surface of breast cancer cells, reducing the suppression of immune cells. In a mouse breast cancer model, the injection of this protein complex effectively reduced sialylation, enhanced immune cell infiltration and activation in breast cancer tissues, and prolonged the survival of the mice [157]. Additionally, chimeric antigen receptor (CAR)-T cell therapy is used with hematologic tumors but remains challenging in solid tumors. The immunosuppressive cellular environment and sialic-acid-rich stroma of solid tumors make it difficult for T cells to infiltrate and activate. However, the addition of sialidase to CAR-T cells targeting Tn antigens in tumors resulted in the degradation of extracellular matrix sialic acid within the tumor microenvironment. This, in turn, facilitated the infiltration of T cells into the tumor and enhanced anti-tumor activity [158].

### 4.3. Siglecs Blockade

As the sialylation–Siglec interaction inhibits immune cell activity, reducing sialylation in tumor tissue helps to mitigate this restriction; however, blocking Siglec binding is a more effective method for achieving this. Anti-Siglec antibodies have the potential to block interactions between Siglec and its ligands, thereby modulating the function of immune cells (Figure 5). For instance, the Siglec-15 antibody NC318, a humanized monoclonal antibody, was shown to effectively block the function of Siglec-15, leading to the restoration of T-cell killing, the cessation of tumor growth, and the prevention of tumor metastasis in mouse models that constitutively express Siglec-15 [81]. Although, the phase I/II clinical studies of NC318 did not make satisfactory progress, this therapy remains a promising treatment for immune rescue [159,160,161]. Siglec-7 and Siglec-9 are also well-studied receptors, and novel antibodies were designed based on these receptors. A method was developed to produce Siglec antibodies by immunizing mice with human-derived Siglec-9 and screening antibody cells enriched from the spleen for clones with a high affinity, resulting in the identification of the 8A1E9 clone. In a humanized mouse model of ovarian cancer, treatment with anti-Siglec-9 effectively reduced tumor volume [162].

The three main methods of blocking the sialylation–Siglecs axis can effectively promote the inhibitory effect of tumor sialylation, thus recovering the killing activity of immune cells, which has a very positive effect on tumor treatment (Figure 5).

## 5. Discussion and Future Perspectives

Monosaccharides are a highly valued source of energy for cells. However, cells have developed an extensive network for synthesizing and processing glycosylation, utilizing glycans to modify essential proteins, lipids, and even nucleic acids on the cell surface. Human exploration of glycans has progressed from the early days of isolating and identifying glycan composition and identifying glycosylated proteins or lipids to the use of high-throughput tools for studying glycosyltransferases and glycomics. These explorations provided the foundations for current research on the global effects of glycosylation in tumor cells from a microenvironmental perspective.

In recent years, the crucial role of glycosylation has received increased recognition, especially negatively charged monosaccharides such as sialic acid. Sialylation has been found to significantly accumulate in tumor cells and tissues, as demonstrated in previous studies. Utilizing high-throughput techniques such as transcriptomics and proteomics, researchers have identified sialyltransferases that contribute to the significant accumulation of sialic acid and have extensively studied their roles in tumor proliferation, apoptosis, metastasis, and resistance to treatment. However, the in-depth characterization of glycosylation remains challenging due to technical limitations. Specifically, intact glycan analysis, particularly at the single-cell level, is still in the stage of early exploration. It is crucial to develop techniques for analyzing glycan modifications at the single-cell level, especially at specific sites on key ligands, to facilitate a better understanding of the role of glycosylation in the tumor microenvironment. Such advancements in technology would greatly enhance our ability to explore the complex interactions between sialylated glycans and immune systems in cancer.

Sialic acid modification acts similar to a cloak, concealing the true identity of the tumor from the immune system and preventing recognition and attacks. Under normal conditions, sialic acid expression is also upregulated during inflammatory responses to protect normal cells from immune cell attacks. However, tumors exploit this property by overexpressing sialic acid to evade immune system surveillance. Despite the explosive growth of research on the sialylation–Siglec axis in recent years, the mechanism by which sialic acid suppresses the immune microenvironment is still not fully understood and requires further investigation. While tumor therapies targeting the sialylation–Siglec axis have been reported, the number of patients who enter the clinic is not substantial. Moreover, the most promising antibody against Siglec-15 was not as effective as expected in the phase II clinic trial [159,160,161], and greater investment is needed to design effective drugs.

## 6. Conclusions

In summary, tumor cells accumulate sialylation in tissues via the coordination of sialyltransferases and sialidases. Sialylation can block receptor and ligand binding at a physical level and actively inhibit immune activation by binding to the Siglec receptor of immune cells, creating an immunosuppressive microenvironment. Blocking the sialylation–Siglec axis in tumor tissues could alleviate the suppression of the immune microenvironment.

## Data Availability

Not applicable.
